# Sex and Age Differences in Default Mode Network Functional Correlation After Traumatic Brain Injury

**DOI:** 10.1093/geroni/igab046.3025

**Published:** 2021-12-17

**Authors:** Anar Amgalan, Alexander Mayer, Michelle Ha, Andrei Irimia

**Affiliations:** 1 University of Southern California, Los Angeles, California, United States; 2 University of Southern California, University of Southern California, California, United States

## Abstract

The extent to which brain functional correlations (FCs) are modulated by age and sex is unknown. We studied default mode network (DMN) FC changes in 136 participants with mild traumatic brain injury (mTBI; 52 females, age range: 19 – 79 years, age μ = 42, age σ = 17; 72 participants younger than 40). Structural and functional magnetic resonance images (MRIs) were acquired ~1 week and ~6 months post-injury; the FreeSurfer Functional Analysis STream (FS-FAST) was used for group-level FC comparisons across sexes and age groups (younger vs. older than 40). FC seeds were two sub-networks of the DMN, M1 and M2, defined by the standard Yeo parcellation scheme. For M1, clusters with significant FC differences across sexes were in the right paracentral lobule, central sulcus, postcentral gyrus, superior frontal gyrus, and precentral sulcus (p = 0.0001), and in the left paracentral lobule and central sulcus (p = 0.022). For M2, clusters spanned the right postcentral gyrus, middle occipital gyrus, transverse occipital sulcus, and central sulcus (p = 0.0001), the left precuneus and inferior parietal lobe (p = 0.0096). Females either exhibited no significant FC change or underwent FC increases. Males underwent significant FC decreases within all clusters, suggesting their increased vulnerability to mTBI-related effects. Clusters whose FCs differed significantly across age groups were localized to the left superior temporal gyrus (p = 0.0078), highlighting the vulnerability of temporal regions to age effects. Future studies should explore the age × sex interaction and uncover the mechanisms for these observed findings.

